# Stigmatizing attitudes toward mental illness among caregivers of patients with mental disorders in China

**DOI:** 10.3389/fpubh.2023.1071954

**Published:** 2023-06-22

**Authors:** Yuzhu Hao, Qiuxia Wu, Xuyi Wang, Yuejiao Ma, Yunfei Wang, Pu Peng, Xin Wang, Qian Yang, Yueheng Liu, Manyun Li, Li He, Qianjin Wang, Yanan Zhou, Tieqiao Liu, Shubao Chen

**Affiliations:** ^1^National Clinical Research Center on Mental Disorders, Department of Psychiatry, The Second Xiangya Hospital, Central South University, Changsha, China; ^2^Department of Psychiatry, Hunan Brain Hospital (Hunan Second People’s Hospital), Changsha, China

**Keywords:** stigma, caregivers, depression, schizophrenia, GAD

## Abstract

**Objective:**

This study aimed to investigate stigmatizing attitudes toward depression, schizophrenia, and general anxiety disorder (GAD) among caregivers of patients with mental disorders in China.

**Methods:**

A cross-sectional study was conducted among 607 caregivers in China, using vignettes that described three mental illnesses. Data on the caregivers’ attitudes and other people’s attitudes toward individuals with mental disorders and their willingness to come in contact with people with mental disorders were collected.

**Results:**

In the three vignettes, caregivers agreed that positive outcomes outnumbered negative outcomes. The top two statements endorsing the stigma were “the person could snap out of the problem” and “people with this problem are dangerous.” In the section for perceived stigma, caregivers in the GAD vignette agreed that most people believed this problem is not a real medical illness, compared to schizophrenia. The rates of the statement endorsing unpredictability were significantly different in the schizophrenia (57.2%) and depression (45.5%) vignette, in comparison to the GAD (45.6%) vignette. For personal stigma, the caregivers tended to avoid people described in the depression vignette more often than in the GAD vignette. The caregivers were most unwilling to let the person described in the vignettes marry into their family, especially in the schizophrenia vignette.

**Conclusion:**

Despite the stigma and desire for social distance associated with schizophrenia, depression, and GAD, caregivers often expect positive outcomes. Actions should be taken to improve caregivers’ knowledge about mental health and reduce the stigma.

## 1. Background

Stigma is defined as a deeply discrediting attribute under certain conditions directed towards those considered as having a lower social standing (1). According to Griffiths et al., stigma can be divided into two dimensions: personal stigma (the individual’s own attitudes toward the stigmatized group) and perceived stigma (the individual’s perception of other people’s attitudes toward the stigmatized group) (2). Stereotypes, prejudice, and discrimination also consist of three vital dimensions of stigma (3). Stereotypes refer to negative attitudes rooted in social life; accepted stereotypes could become prejudices and are frequently accompanied by negative emotional reactions (4). This inevitably leads to avoidance and social distancing, resulting in discrimination (3). Stigma always represents the consequences of a series of processes: people’s conceptualizations of the meaning of suffering from a mental illness, labeling people suffering from a mental illness as “different” with characteristics that are undesired by others and considering them as lacking in intelligence and competence (5). These lead to stigmatized people being devalued and discriminated against (5).

Stigmatizing attitudes toward mental disorders are widespread and evident across all cultures and societies. Individuals with different kinds of mental disorders reported experiencing discrimination (6–8), which is always linked to serious consequences such as a delay in seeking treatment and poor adherence to psychosocial (9, 10) and pharmacological (11, 12) treatment, which may further lead to a poor prognosis. Last but not least, stigma can reduce one’s sense of hope and self-esteem, which may lead to a series of undesirable consequences related to recovery, including social avoidance, depressive symptoms, a preference for using avoidant coping strategies (13), and poorer quality of life (5).

Mental illnesses are common around the world and constitute a burden on people and society. In China, the lifetime prevalence of mental disorders is 16.6% (14). This has created an enormous economic burden, including the high cost of treatment and return to society (15). Though mental illnesses have created a heavy burden on families, not every patient has received timely and effective treatment. A growing number of research in many countries has shown that stigma is a crucial treatment barrier that leads to a delay in seeking treatment (16–20). Those who are afraid of being stigmatized because of their mental problems may not seek professional help in time (21), which may worsen their mental problems. A community-based study conducted in China confirmed that a huge proportion of the general population perceived that the public held strongly negative attitudes toward people with mental illness (22). These attitudes may lead to intense perceived stigma and further result in a delay in seeking treatment. Early intervention seems to ensure a more favorable prognosis for people with mental illness (23). Summarizing the above facts, stigma toward mental disorders may have serious effects on the disease itself, patients’ life, their family, and the whole of society. Such problems need urgent attention and action.

There is a scarcity of research on mental health-related stigma in the Chinese context. Several studies among different populations such as the general public, psychiatrists, non-mental health professionals, and caregivers of patients with mental disorders in China have shown poor mental health literacy (24–28), which may be one of the reasons linked to mental health-related stigma. Surveys among the general public in China have found that many participants believed that most individuals reported personal and perceived stigma and had a strong desire to keep social distance from people with mental disorders (19, 22). Stigma related-studies conducted in China usually focused on schizophrenia and depression but neglected another common mental disorder—anxiety disorder. The high prevalence, chronicity, and burden of anxiety disorders (29), particularly generalized anxiety disorder (GAD), necessitates research into the anxiety-related stigma. Besides, before evaluating participants’ stigma level, most of these studies used only one word instead of a vignette describing the disease, which inevitably leads to certain deviations.

In China, previous studies explored the perceived stigma of caregivers who spend much time and effort taking care of the patients, but few studies investigated caregivers’ stigma of mental illness. The disease itself, the challenges of caring, as well as the cultural and social negative connotations tend to bring many difficulties to caregivers (30). Caregivers of people with mental illness play a key role in family resilience [the capability to adapt to adverse situations (31)], and the stigmatizing attitudes that they may have is one of the barriers to family resilience (32).

Given that caregivers’ stigmatizing attitudes occupy an important role not only in the treatment of patients but also in the prognosis, we conducted the present study to deepen our understanding of their stigma and social distance toward people with depression, schizophrenia, and GAD, and to further compare the stigma levels between these three mental disorders. We hypothesized that caregivers’ stigmatizing attitude (including social distance) toward schizophrenia would be the highest, and the stigmatizing attitude toward GAD would be the lowest.

## 2. Methods

### 2.1. Sample and design

This was a cross-sectional and vignette-based study that followed a cluster convenience sampling method. The survey was conducted from January to December 2014. The rates of correct identification of mental health among caregivers were taken into consideration for sample size calculation. We estimated the range of 15% for our sample size calculation, with a precision of 5%; we set a 5% marginal error and took into consideration a 10% non-response rate. Therefore, the calculated sample size is 215 for each vignette and 645 for three vignettes. We delivered 645 questionnaires, and 607 caregivers completed the questionnaires. The response rate was 94.1%.

All participants were recruited from the Department of Psychiatry in a general hospital located in Changsha, China, which is the capital city of the Hunan province. The data for this article was drawn from part of our previous research. Inclusion criteria were that participants had to be at least 18 years old and living with and taking care of people diagnosed with any mental disorders. If there was more than one caregiver of a patient, we chose the main one who volunteered to complete the questionnaire. We excluded those who were diagnosed with mental disorders themselves and those who refused to participate in the study.

Signed informed consent was obtained from each participant before completing the questionnaires. All participants completed the questionnaire independently, and it took them 20–30 min to complete. If the participants had difficulty reading the questionnaire due to poor sight or low literacy, investigators who were graduate students read the items verbally and recorded the response.

### 2.2. Survey questionnaires

In our study, the questionnaires and vignettes were adapted from the questionnaire developed by Jorm et al. (33). After providing the consent form, each participant was asked to provide demographic information including gender, age, marital status, education level, occupation, and relationship with the patients. Then, they were randomly assigned one of the three vignettes describing schizophrenia, depression, or GAD. The symptoms in each vignette were designed to meet the diagnostic criteria for schizophrenia, depression, or GAD in accordance with the Diagnostic and Statistical Manual of Mental Disorders (4th edition, DSM-4) and the International Statistical Classification of Diseases and Related Health Problems (10th revision, ICD-10).

Participants were asked to answer a series of mental health literacy-related questions after they were presented with the vignette, including the most likely diagnosis, likely effective and harmless interventions and medications, and likely causes and risk factors of the disease. The data associated with these questions was reported in our previous article (26). Following the questions mentioned above, participants’ attitudes toward the likely outcome of the person described in the vignette after receiving helpful interventions, participants’ stigmatizing attitudes, participants’ desire for social distance, and the ways that participants obtained information about mental health were also asked about. This paper mainly focuses on participants’ stigmatizing attitudes and desire for social distance toward individuals with mental disorders.

#### 2.2.1. Personal and perceived stigma

Participants’ stigmatizing attitudes were assessed using an 18-item scale adapted from the Depression Stigma Scale which includes two subscales (34). One of the subscales asks about participants’ attitudes toward the person described in the vignettes, which reflects participants’ personal stigma. The other subscale asks participants’ beliefs about other people’s attitudes toward the person described in the vignettes, which reflects participants’ perceived stigma. The contents of both scales are similar; the difference is that one aims at personal attitudes and the other aims at the perceived attitudes of others. Each subscale includes nine items using a 5-point Likert scale, which ranges from four points (strongly agree) to zero points (strongly disagree). Higher points represent greater personal or perceived stigma. The two choices (“strongly agree” or “agree”) for each item are usually combined to represent the presence of stigma or perceived stigma (2). The versions used in our study have shown great reliability and validity in China (35). The internal consistency was corroborated by a Cronbach’s alpha of 0.785 for the schizophrenia vignette, 0.740 for the depression vignette, and 0.754 for the GAD vignette.

#### 2.2.2. The desire for social distance

A 5-item scale developed by Link to evaluate the willingness to make contact with the person described in the vignette was used to access participants’ desire for social distance (36). Each item was rated on a 4-point scale ranging from “definitely willing” to “definitely unwilling.” The versions used in our study have shown great reliability and validity in China (35). The Cronbach’s alpha of this scale in this study was 0.886 for the schizophrenia vignette, 0.896 for the depression vignette, and 0.763 for the GAD vignette, which showed high reliability. Higher scores indicate a greater desire for social distance, otherwise known as less social acceptance.

### 2.3. Statistical analysis

All data were entered by double-entry strategy in EpiData version 3.1 (EpiData Association, Odense, Funen, Denmark). All data analyses were conducted in R 3.6.1 within R studio 1.2.5001 (37). Descriptive analyses using median (interquartile range) for skewed variables and frequency (percentage) for categorical variables were used for demographic data. Then, we computed the percentage and 95% confidence interval (CI) for each endorsed statement. Chi-square tests and Kruskal-Wallis tests were performed to investigate whether there were any demographic differences among the vignettes. The answers for “agree” and “strongly agree” of the stigma items, and “definitely unwilling” and “probably unwilling” categories of the social distance items were combined separately for further analyses. For social distance, we also calculated the mean and 95% CI of social distance scores to further understand the whole level. For each item in all scales, including mean scores of social distances, we used Pearson’s Chi-square test to investigate whether any significant difference exists among the three vignettes and calculated the odd rate (OR) and 95% CI.

## 3. Results

A total of 607 questionnaires were collected and included in the final sample, with 201 for the schizophrenia vignette, 202 for the depression vignette, and 204 for the GAD vignette, respectively. As shown in Table 1, of all caregivers, 56.3% were female, 88.1% were married, and the median age was 42 years old. No significant difference was observed among participants’ gender, age, educational level, and occupations in the three vignettes (*p* > 0.05). There were significant differences in marital status and relationship with the patients between the GAD and schizophrenia vignettes (*p* < 0.05).

**Table 1:** Demographic characteristics of the caregivers.

Caregivers’ characteristics		Total		GAD vignette (*n* = 204)		Schizophrenia vignette (*n* = 201)		Depression vignette (*n* = 202)		*p*-value
		*n*	%	*n*	%	*n*	%	*n*	%	
Gender	Male	265	43.7	98	48.0	82	40.8	85	42.1	0.291
	Female	342	56.3	106	52.0	119	59.2	117	57.9	
Age (year)*		42.0	33.0–48.0	41.0	33.0–48.0	41.5	36.0–48.0	40.5	31.3–48.0	0.695
Marital status	Married	535	88.1	181	88.7	176	87.6	178	88.1	0.034**
	Single	64	10.6	23	11.3	18	8.9	23	11.4	
	Other status	8	1.3	0	0	7	3.5	1	0.5	
Education level	Primary school education and below	34	5.6	8	3.9	13	6.4	13	6.4	0.207
	Junior high school education	197	32.4	62	30.4	75	37.3	60	29.7	
	Senior high school education	157	25.9	48	23.5	53	26.4	56	27.7	
	Bachelor’s degree	196	32.3	80	39.2	53	26.4	63	31.2	
	Master’s degree and above	23	3.8	6	3.0	7	3.5	10	5.0	
Occupations	Workers	82	13.5	28	13.7	26	12.9	28	13.9	0.362
	Farmers	185	30.5	54	26.5	72	35.8	59	29.2	
	Cadres	63	10.4	22	10.8	25	12.4	16	7.9	
	Intellectuals	69	11.3	23	11.3	19	9.5	27	13.4	
	Other occupations	208	34.3	77	27.7	59	29.4	72	35.6	
Relationship with the patients	Father	105	17.3	27	13.2	39	19.4	39	19.3	0.047**
	Mother	160	26.4	44	21.6	65	32.3	51	25.2	
	Siblings	79	13.0	31	15.2	23	11.5	25	12.4	
	Other relationship	263	43.4	102	50.0	74	36.8	87	43.1	

*Data descriptive with median (interquartile range). **p < 0.05, and *post hoc* multiple comparisons found a significant difference between the GAD and schizophrenia vignette (*p* < 0.05).

### 3.1. Beliefs about likely outcomes

We asked all caregivers to select a likely outcome for the person described in the vignette after receiving helpful interventions. As described in Table 2, the caregivers thought “being in a bad relationship” would be the most likely negative result for the person described in the three vignettes (46.8% for the schizophrenia vignette, 37.1% for the depression vignette, and 42.2% for the GAD vignette). The rate of caregivers who thought “a propensity for violence” was the most likely scenario was higher in the schizophrenia vignette than in the depression vignette (*p* = 0.005, OR:1.99, [1.22, 3.67]). Caregivers in all three vignettes endorsed the statement that “being better and understanding the feelings of others” would be the best positive result for the person described in the vignettes after receiving treatment (57.2% for the schizophrenia vignette, 67.8% for the depression vignette, and 62.3% for the GAD vignette). The endorsement rate between schizophrenia and depression vignettes was significantly different (*p* < 0.05, OR:1.99, [1.22, 3.67]). Caregivers’ endorsement rate of positive results was slightly higher than negative results in all vignettes. In the schizophrenia vignette, the rates of all items with negative results were the highest; the rates of items except for “being an artistic person” in the positive results were the lowest in the schizophrenia vignette.

**Table 2:** Percentage (and 95% CI) of the caregivers selecting each outcome as a most likely scenario toward the person in the vignette after receiving the helpful intervention.

	GAD vignette (*n* = 204)		Schizophrenia vignette (*n* = 201)		Depression vignette (*n* = 202)		*p*-value
	*n*	%	*n*	%	*n*	%	
Negative results
A propensity for violence	47	23.0 (17.6, 29.5)	62	30.8 (24.6, 37.8)	37	18.3 (13.4,24.5)	0.012^A^
A propensity for alcoholism	31	15.2 (10.7, 21.0)	39	19.4 (14.3, 25.7)	37	18.3 (13.4,24.5)	0.514
A propensity for using drugs	23	11.3 (7.4, 16.6)	32	15.9 (11.3, 21.9)	26	12.9 (8.7,18.5)	0.378
Being in a bad relationship	86	42.2 (35.4, 49.3)	94	46.8 (39.6, 53.9)	75	37.1 (30.5,44.2)	0.146
Suicidal tendency	58	28.4 (22.5, 35.2)	71	35.3 (28.8, 42.4)	58	28.7 (22.7,35.6)	0.237
Positive results
Being better and understanding the feelings of others	127	62.3 (55.2, 68.9)	115	57.2 (50.1, 64.1)	137	67.8 (60.8,74.1)	0.089^B^
Being in a happy marriage	115	56.4 (49.3, 63.2)	97	48.3 (41.2, 55.4)	117	57.9 (50.8,64.8)	0.112
Being a caring father or mother	118	57.8 (50.7, 64.6)	110	54.7 (47.6, 61.7)	125	61.9 (54.8,68.5)	0.900
Being an efficient employee	82	40.2 (33.5, 47.3)	77	38.3 (31.6, 45.4)	92	45.5 (38.6,52.7)	0.310
Being a creative person	66	32.6 (26.1, 39.3)	67	33.3 (27.0, 40.4)	80	39.6 (32.9,46.7)	0.253
Being an artistic person	49	24.0 (18.5, 30.6)	55	27.4 (21.4, 34.2)	59	29.2 (23.1,30.1)	0.490

A Schizophrenia: depression, *p* = 0.005, OR: 1.99, [1.22, 3.67].

B Schizophrenia: depression, *p* = 0.012, OR: 1.61, [1.22, 2.34].

### 3.2. Personal and perceived stigma

Table 3 revealed to us the percentage (and 95% CI) of caregivers who “agree” or “strongly agree” with statements about their personal stigma and perceived stigma toward the person described in the vignettes. In the GAD vignette, the highest three endorsements were for the statements “the person could snap out of the problem,” “people with this problem are dangerous,” and “this kind of problem is a sign of personal weakness” for personal and perceived stigma. For perceived stigma, the caregivers agreed that most other people believed “this kind of problem is not a real medical illness” compared to schizophrenia (GAD: schizophrenia, *p* < 0.05, OR:1.71, [1.06, and 2.79]). In the schizophrenia vignette, “people with this problem are dangerous,” “the person could snap out of the problem,” and “people with this problem are unpredictable” were the three statements with the highest endorsements. The rates of agreement with the statement “people with this problem are dangerous” were the highest for the schizophrenia vignette compared to the other two vignettes. For perceived stigma, the rates of the statement “people with this problem are unpredictable” were significantly different from the schizophrenia and depression vignette [(schizophrenia: depression, *p* < 0.05, OR:1.60, [1.06, 2.42]), GAD and schizophrenia vignette (GAD: schizophrenia, *p* < 0.05, OR: 0.63, [0.42, 0.96])]. The statements with the top three highest endorsements for the depression vignette for personal and perceived stigma were “the person could snap out of the problem,” “people with this problem are dangerous,” and “I would not vote for a politician with this problem.” For personal stigma, the caregivers were more likely to believe the statement “avoid people with this problem” in the depression vignette than in the GAD vignette (GAD: depression, *p* = 0.010, OR:0.47, [0.25, 0.87]).

**Table 3:** Personal and perceived stigma of the caregivers.

	GAD vignette (*n* = 204)		Schizophrenia vignette (*n* = 201)		Depression vignette (*n* = 202)		*p* value
**Personal stigma**	*n*	%	*n*	%	*n*	%	
1. The person could snap out of the problem	155	76.0 (69.4, 81.5)	143	71.1 (64.3, 77.2)	161	79.7 (73.4, 84.9)	0.134
2. This kind of problem is a sign of personal weakness	94	46.1 (39.1, 53.2)	80	39.8 (33.0, 46.9)	82	40.6 (33.8, 47.7)	0.378
3. This kind of problem is not a real medical illness	46	22.5 (17.1, 29.0)	32	15.9 (11.3, 21.9)	34	16.8 (12.1, 22.9)	0.175
4. People with this problem are dangerous	131	64.2 (57.1, 70.7)	159	79.1 (72.6, 84.3)	139	68.8 (61.9, 75.0)	0.003^C^
5. Avoid people with this problem	20	9.8 (6.2, 14.9)	31	15.4 (10.8, 21.3)	38	18.8 (13.8, 25.0)	0.035^D^
6. People with this problem are unpredictable	85	41.7 (34.9, 48.8)	98	48.7 (41.6, 55.9)	90	44.6 (37.6, 51.7)	0.354
7. If I had this problem, I would not tell anyone	46	22.5 (17.1, 29.0)	50	24.8 (19.2, 31.5)	37	18.3 (13.3, 24.5)	0.272
8. I would not employ someone with this problem	72	35.3 (28.8, 42.3)	89	44.3 (37.3, 51.4)	79	39.1 (32.4, 46.2)	0.179
9. I would not vote for a politician with this problem	92	45.1 (38.2, 52.2)	91	45.3 (38.3, 52.4)	91	45.0 (38.1, 52.2)	0.999
Perceived stigma
1. The person could snap out of the problem	157	77.0 (70.5, 82.4)	141	70.1 (63.2, 76.3)	159	78.7 (72.3, 84.0)	0.092
2. This kind of problem is a sign of personal weakness	84	41.1 (34.4, 48.3)	97	48.3 (41.2, 55.4)	82	40.6 (33.8, 47.7)	0.224
3. This kind of problem is not a real medical illness	61	29.9 (23.8, 36.8)	40	19.9 (14.7, 26.2)	48	23.8 (18.2, 30.4)	0.062^E^
4. People with this problem are dangerous	118	57.8 (50.7, 64.6)	143	71.1 (64.3, 77.2)	106	52.5 (45.4, 59.5)	0.000^F^
5. Avoid people with this problem	47	23.0 (17.6, 29.5)	52	25.9 (20.1, 32.6)	35	17.3 (12.5, 23.4)	0.109
6. People with this problem are unpredictable	93	45.6 (38.7, 52.7)	115	57.2 (50.1, 64.1)	92	45.5 (38.6, 52.7)	0.026^G^
7. If I had this problem, I would not tell anyone	61	29.9 (23.8, 36.8)	46	22.9 (17.4, 29.4)	45	22.3 (16.9, 28.8)	0.143
8. I would not employ someone with this problem	91	44.6 (37.7, 51.7)	72	35.8 (29.3, 42.9)	85	42.1 (35.2, 49.2)	0.181
9. I would not vote for a politician with this problem	98	48.0 (41.0, 55.1)	92	45.8 (38.8, 52.9)	97	48.0 (41.0, 55.1)	0.872

C Schizophrenia: depression, *p* = 0.025, OR: 1.71, [1.07, 2.77]. GAD: schizophrenia, *p* = 0.001, OR: 0.47, [0.29, 0.76].

D GAD: depression, *p* = 0.010, OR: 0.47, [0.25, 0.87].

E GAD: schizophrenia, *p* = 0.027, OR: 1.71, [1.06, 2.79].

F GAD: schizophrenia, *p* = 0.007, OR: 0.56, [0.36, 0.86]. Schizophrenia: depression, *p* = 0.000, OR: 2.23, [1.45, 3.44].

G Schizophrenia: depression, *p* = 0.024, OR: 1.60, [1.06, 2.42]. GAD: schizophrenia, *p* = 0.025, OR: 0.63, [0.42, 0.96].

### 3.3. Social distance

The caregivers’ endorsements of probably or definitely unwilling to have contact with the person (social distance) described in the vignette are shown in Table 4. Among all items, a majority of the caregivers were mostly unwilling to let the person described in the vignette marry into their families (>65%), especially in the schizophrenia vignette (77.1%). The desire for social distance was the least likely for the item “spending the evening socializing.” We also calculated the mean social distance scores of the three vignettes. The mean social distance score in the depression vignette was 10.84 (95%CI: [10.33, 11.34]). A desire for social distance was highest for the schizophrenia vignette (mean: 11.67, 95%CI: [11.15, 12.18]) and lowest for the GAD vignette (mean: 9.64, 95%CI: [9.25, 10.03]), and the difference between the three vignettes was not significant (*p* > 0.05).

**Table 4:** The desire for a social distance of the caregivers.

Social interactions	GAD vignette (*n* = 204)		Schizophrenia vignette (*n* = 201)		Depression vignette (*n* = 202)		*p*-value
	*n*	%	*n*	%	*n*	%	
1. Live next door	63	30.9 (24.7, 37.8)	69	34.3 (27.9, 41.4)	61	30.2 (24.1, 37.1)	0.634
2. Spend the evening socializing	42	20.6 (15.4, 26.9)	55	27.4 (21.4, 34.2)	41	20.3 (15.2, 26.6)	0.160
3. Make friends	51	25.0 (19.3, 31.6)	60	29.9 (23.7, 36.8)	47	23.3 (17.8, 29.8)	0.296
4. Work closely	82	40.2 (33.5, 47.3)	90	44.8 (37.8, 51.9)	72	35.6 (29.1, 42.7)	0.174
5. Marry into the family	142	69.6 (62.7, 75.7)	155	77.1 (70.6, 82.6)	133	65.8 (58.8, 72.3)	0.040^H^

H Schizophrenia: depression, *p* = 0.016, OR: 1.75, [1.10, 2.78].

### 3.4. The caregivers’ usual sources to get mental health knowledge

As a whole, the caregivers reported that the most common way to learn about mental health was through websites (44%), followed by other people’s explanations (37.4%). The least common way was through newspapers (14.8%). Detailed information can be found in Supplementary Table S1.

## 4. Discussion

This survey was conducted among caregivers of patients with mental illness in China to investigate their beliefs about likely outcomes, stigmatizing attitudes, and social distance toward individuals diagnosed with depression, schizophrenia, and GAD. Broadly speaking, the survey showed that the caregivers’ beliefs about positive outcomes were relatively high in all vignettes. Consistent with our hypothesis, individuals described in the schizophrenia vignette were thought to have more negative outcomes and less positive outcomes, and individuals described in the depression vignette were believed to have positive outcomes. For personal and perceived stigma, caregivers tended to agree or strongly agree with the statements, “the person could snap out of the problem” and “people with this problem are dangerous” in all vignettes. Beliefs about dangerousness and the description of unpredictability were highest in the schizophrenia vignette. GAD was the least to be thought of as a real medical illness compared to other disorders. The caregivers tended to avoid the person described as depressed. The desire for social distance was universally higher for schizophrenia than for other diseases. More people were reluctant to let a person with schizophrenia marry into their family than those with depression. The most common source of information caregivers used to obtain information on mental health was websites.

We found that agreements on positive results were higher than negative results in all vignettes. On account of their relatives’ mental illness, the caregivers may be more active to look into this kind of disease and tend to believe in a positive future. This was in accordance with the results of the stigma that, though the caregivers think the individual described in the vignette was dangerous, they still believe the individual could snap out of the problem. The caregivers of individuals with mental illness always experienced stigma themselves, which even led to the occurrence of psychological morbidity (38, 39). Hence, the positive future of the patients with mental illness may represent their hope for a bright future for themselves. As for GAD, the reason for positive outcomes may be that they did not regard GAD as a real medical disease, which can also be found in our published study (40). However, the latest epidemiological survey data in China found that the prevalence of anxiety disorder over the past 12 months and lifetime prevalence were higher than for depression and schizophrenia (14). These data point to the need to improve the public’s knowledge about GAD, given that anxiety disorder is a much more common mental disorder that severely affects an individual’s work and life. Our research found that participants thought patients with depression could better understand others’ feelings than patients with schizophrenia. This could be, in most people’s opinion, because patients with depression may experience more emotional pain, hence they are more sensitive to others’ feelings.

For the negative results, for personal and perceived stigma, the caregivers had displayed the same opinion that the dangerousness and the description of the unpredictability of schizophrenia were higher than the other two diseases. These stereotypes of patients with schizophrenia frequently led to fear and uncertainty (41), which were common negative attitudes held by the general public toward patients internationally (42). One possible interpretation was that the caregivers had witnessed or experienced the episode of the patient at the height of a psychotic episode. They are afraid of the same episode or a worse one. Another reason for the belief in dangerousness may stem from biased reports by the media. Media reports of violence committed by people with mental illnesses have been suggested as a potent contributor to aggravating public stigma (43), in which the portrayal is always stereotypical and prejudiced. Our study demonstrated that the most common source of mental health knowledge was through websites—a new style and common media in modern society. It revealed the significance of media in guiding the public correctly. To avoid increased public stigma and discrimination of people with mental illness, the standardization and confidentiality of correlative media reporting should be given more attention.

Current findings are also in accord with those of other studies that report a higher level of stigma toward schizophrenia than other mental illnesses (2, 20, 42, 44–46), and the results indicated that the item “people with this problem are unpredictable” for perceived stigma was agreed or strongly agreed by more caregivers in the schizophrenia vignette than in others. Like the belief of the dangerousness of these patients, this could bring fear and uncertainty. As a result, this may lead to a high rate of unemployment. This study also verified a high proportion of unwillingness to employ people with mental illness. A study conducted in China shows that people with schizophrenia are either unemployed or work in agriculture or fishing (47). In these kinds of agrarian jobs, stigma may be mitigated by the more informal hiring practices based on kinship networks and fewer formal requirements for employment (48). For personal stigma, participants deemed that people should avoid patients with depression more than patients diagnosed with GAD. In most people’s opinion, patients with depression have more tendency to hurt themselves than patients with GAD. Research found that suicidal behaviors were very common in patients with depression in China (49). In China, most people would choose to avoid these patients to stay away from those patients, don’t get into trouble and be on peace.

In line with previous studies on stigmatizing attitudes, most questionnaire items assessing perceived stigma received a much higher endorsement than those assessing personal stigma except for beliefs in dangerousness. A more likely explanation is the consequence of social desirability. People tend to give socially desired answers to questions about controversial issues to maintain harmony (50, 51). Some people in China influenced by traditional Chinese culture, such as Confucianism and Taoism, are conservative about making self-statements, and they prefer to endorse the stigmatizing beliefs of others than to admit their own. Maintaining social harmony is believed to be so important that it may take precedence over expressing one’s opinions and values. People tend to accept public stigma toward mental illness, and the role of acceptance of fate and destiny promotes public stigma. Another reason for a higher perceived stigma may be that public stigma was likely to extend to family members (1). Caregivers of the patients may be thought to have a family history of the mental illness and may likewise have the possibility of having the mental illness.

Similar to personal and perceived stigma, the desire for social distance by caregivers was higher in the schizophrenia vignette than in the GAD and depression vignette, which may also be a reflection of participants’ belief in the dangerousness and unpredictability of schizophrenia. In line with previous research, people were most reluctant to allow patients with mental illness to marry into their families. Compared with other behaviors, marrying into the family was the most intimate one and may have a significant impact on their family members. Beliefs in the neurochemical and general biogenetic causation of mental illness may cause people to worry that the illness may be passed on to subsequent generations (52). Meta-analysis showed that these beliefs were associated with a desire for social distance from patients with schizophrenia; they are also associated with an increased belief in dangerousness for all mental disorders (53).

Exposure to people with mental illnesses has previously been found to be associated with lower personal stigma (54, 55) and higher perceived stigma (54) toward people with mental illnesses because diagnosis leads to a greater understanding of the disorders and less stigmatizing viewpoints about people with these disorders. However, our study found there was still a high level of stigma among caregivers of patients with mental illness. In a study in Hanoi, Vietnam, the authors believed that there was a higher level of stigma among the public than among people who had contact with patients with mental illness (56). Our study among non-mental health professionals also supported this inference (20), but a study in Hong Kong found that there was a comparable stigma among family members and community residents, and they attribute this to the non-significant difference in knowledge and contact quality (57). Meanwhile, caregivers could be harmed by stigma themselves (58). Stigma is one of the significant factors of distress among family caregivers of persons living with schizophrenia (59).

Therefore, given the high level of stigma and desire for social distance and the adverse impact brought by stigma, more interventions need to be done to decrease the stigma. There are some anti-stigma campaigns conducted in many countries to eradicate the public’s stigma toward patients with mental disorders, thus further improving patients’ treatment compliance and recovery prognosis (16, 60, 61). Programs that support people with mental illness to cope with stigma could improve suicide prevention (62). A systematic review has proven the positive effect of the anti-stigma intervention on stigma and social distance (63). Our study found that people often get mental health-related knowledge from websites and television, thus relevant anti-stigma campaigns should be carried out through these channels. More niche targeting suitable for the Chinese population and widespread projects are yet to be carried out. Lack of awareness and knowledge about mental illness is frequently identified as a cause of mental illness stigma (64).

## 5. Conclusion

In conclusion, the current findings suggest that in China, there is a high level of stigma and desire for social distance among caregivers of patients with mental illnesses like schizophrenia, depression, and GAD, especially in schizophrenia. Though the caregivers thought patients with these conditions were dangerous, their beliefs about the likely outcomes were positive. Perceived discrimination prevents individuals with mental illness from seeking help and accessing available resources. Our study provides valuable implications for further effective targeted interventions to reduce the stigma toward patients with mental illness. Actions should be taken by the government to provide publicity campaigns among caregivers to improve knowledge about mental health, awareness of how to seek help and treatment, and reduce the stigma to promote the development of social support.

## 6. Limitation

The result of the current study should be considered in light of the following limitations. Firstly, this study was limited by cross-sectional data collection, and we were not able to evaluate how the caregivers’ stigma influences the patients’ rehabilitation. A longitudinal study should be carried out to better understand the influence of stigma. Moreover, individuals who completed the study have subtle differences in marital status and relationship with the patients. This may have limited the representativeness of the sample and the generalizability of the study findings. Note that participants from this study were recruited from only one general hospital. The selected and small sample may not represent all caregivers of mental health patients in China. Future research needs to determine how these findings play out in other geographic areas of China, as well as internationally.

## Data availability statement

The raw data supporting the conclusions of this article will be made available by the authors, without undue reservation.

## Ethics statement

Ethical approval was obtained from the Ethics Committee of the Second Xiangya Hospital, Central South University. All participants were informed of the purpose of the study and provided written informed consent for the study.

## Author contributions

TL and SC were responsible for the study design, manuscript preparation, and revision. YH undertook the statistical analysis and wrote the first draft of the manuscript. QiuW and XuW were responsible for writing the protocol and revising manuscript drafts. YM, YW, PP, XiW, QY, YL, ML, LH, QiaW, and YZ were responsible for data collection. All authors contributed to the article and approved the submitted version.

## Funding

This study was supported by the Provincial Natural Science Foundation of Hunan (grant no. 2020JJ4795 to TL) and the Provincial Young Natural Science Foundation of Hunan (grant no. 2022JJ40677 to SC).

## Conflict of interest

The authors declare that the research was conducted in the absence of any commercial or financial relationships that could be construed as a potential conflict of interest.

## Publisher’s note

All claims expressed in this article are solely those of the authors and do not necessarily represent those of their affiliated organizations, or those of the publisher, the editors and the reviewers. Any product that may be evaluated in this article, or claim that may be made by its manufacturer, is not guaranteed or endorsed by the publisher.
