# Supplementary material for: Stigmatizing attitudes toward mental illness among caregivers of patients with mental disorders in China
Source: Front Public Health. 2023 Jun 22;11:1071954. doi: 10.3389/fpubh.2023.1071954 (PMC10325790; doi:10.3389/fpubh.2023.1071954)
Supplement: Supplementary file 1 [file Table_1.DOCX]

**Article**: Stigmatizing attitude towards mental illness among caregivers of patients with mental disorders in China

Supplementary material

Table S1. Usual sources to get mental health related knowledge.

| How do you usually learn about mental health issues? | Total | | GAD vignette(n=204) | | Schizophrenia vignette(n=201) | | Depression vignette(n=202) | | P value |
| --- | --- | --- | --- | --- | --- | --- | --- | --- | --- |
|  | n | % | n | % | n | % | n | % |  |
| Newspaper | 90 | 14.8 | 32 | 15.7(11.1,21.6) | 30 | 14.9(10.4,20.8) | 28 | 13.9(9.6,19.6) | 0.175 |
| Television | 220 | 36.2 | 84 | 41.2(34.4,48.3) | 71 | 35.3(28.8,42.4) | 65 | 32.1(25.9,39.2) |  |
| Websites | 267 | 44.0 | 87 | 42.6(35.8,49.8) | 80 | 39.8(33.0,46.9) | 100 | 49.5(42.4,56.6) |  |
| Books | 167 | 27.5 | 66 | 32.4(26.1,39.3) | 43 | 21.4(16.1,27.8) | 58 | 28.7(22.7,35.6) |  |
| Other people’s explanations | 227 | 37.4 | 77 | 37.7(31.3,44.8) | 81 | 40.3(33.5,47.5) | 69 | 34.2(27.7,41.2) |  |
| Other | 5 | 0.8 | 4 | 2.0(0.6,5.3) | 1 | 0.5(0.0,3.2) | 0 | 0 |  |
